# Natural Epigenetic Protection against the I-factor, a Drosophila LINE Retrotransposon, by Remnants of Ancestral Invasions

**DOI:** 10.1371/journal.pone.0000304

**Published:** 2007-03-21

**Authors:** Xavier Dramard, Thierry Heidmann, Silke Jensen

**Affiliations:** Unité des Rétrovirus Endogènes et Eléments Rétroïdes des Eucaryotes Supérieurs Centre National de la Recherche Scientifique (CNRS) UMR 8122, Institut Gustave Roussy, Villejuif, France; University of Munich, Germany

## Abstract

Transposable elements are major components of most eukaryotic genomes. Such sequences are generally defective for transposition and have little or no coding capacity. Because transposition can be highly mutagenic, mobile elements that remain functional are tightly repressed in all living species. Drosophila pericentromeric heterochromatin naturally contains transposition-defective, non-coding derivatives of a LINE retrotransposon related to the *I*-factor. The *I*-factor is a good model to study the regulation of transposition *in vivo* because, under specific conditions, current functional copies of this mobile element can transpose at high frequency, specifically in female germ cells, with deleterious effects including female sterility. However, this high transpositional activity becomes spontaneously repressed upon ageing or heat treatment, by a maternally transmitted, transgenerational epigenetic mechanism of unknown nature. We have analyzed, by quantitative real time RT-PCR, the RNA profile of the transposition-defective *I*-related sequences, in the Drosophila ovary during ageing and upon heat treatment, and also in female somatic tissues and in males, which are not permissive for *I*-factor transposition. We found evidence for a role of transcripts from these ancestral remnants in the natural epigenetic protection of the *Drosophila melanogaster* genome against the deleterious effects of new invasions by functional *I*-factors. These results provide a molecular basis for a probably widespread natural protection against transposable elements by persisting vestiges of ancient invasions.

## Introduction

In the course of evolution, transposable elements have accumulated in the genome of eukaryotes, where they can account for up to 80% of the DNA [1]. Most of these sequences have lost their ability to transpose. They are now stable components of the genomes. The copies of mobile elements that remain functional are severely repressed by their host, possibly as a biological requisite for genomic stability of species and individuals, since high levels of transposition would result in the accumulation of detrimental insertional mutations and genome rearrangements. The molecular mechanisms involved in this “taming” process are far from being understood, but there is strong evidence that RNA interference (RNAi) [2]–[5] affects the activity of several mobile elements [6]–[8], notably in Drosophila (reviewed in reference [9]).

The Drosophila *I*-factor (FlyBase GeneID number FBGn0001249) belongs to the LINE (Long Interspersed Nucleotidic Element) superfamily [10], which represents the major class of transposable elements in mammals (about 20% of the human genome) [11]. The *I*-factor transposes in a replicative manner, through the reverse transcription of its full-length RNA [12], [13], which encodes the proteins necessary for its mobility. With respect to *I*-factors, all *Drosophila melanogaster* strains fall into two categories named “inducer” and “reactive”. Inducer strains contain transpositionally competent *I*-factors, initially acquired following an invasion of wild flies in the course of the twentieth century. Under normal conditions, these functional copies do not transpose at a detectable rate. Reactive strains lack such functional elements because they had been sequestered in laboratories before the recent invasion mentioned above [14]. However, both categories of strains naturally display a similar pattern of *I*-related elements (*I*-REs) scattered in the pericentromeric heterochromatin of all chromosomes [15], [16]. These sequences are the vestiges of an ancient invasion of the Drosophila genome by a transposable element homologous to the currently active *I*-factor. Today's *I*-REs are non-coding sequences that have lost their ability to transpose due to the accumulation of mutations. These sequences still display 91–95% nucleotide identity with each other and with the functional *I*-factor [17], [18].

The *I*-factor is a powerful tool to study transposon/host interactions *in vivo* because its transposition can be triggered experimentally, using a so-called “dysgenic” cross between reactive females and inducer males. Indeed, the paternal transmission of functional copies of the transposon via crossing with reactive females harboring a “virgin” genome, devoid of such elements, results in a high-frequency transposition detected exclusively in the germ-line of the F1 female progeny (named “SF” for “Stérilité Femelle”). *I*-factor transposition is associated with a high mutation rate, chromosomal non-disjunction, chromosome rearrangements, and female sterility (a syndrome referred to as I-R hybrid dysgenesis) [14], [19]. However, the level of *I*-factor activity may vary, and the ensuing fertility level can be measured, as the percentage of hatching eggs laid by the SF females (crossed with their brothers). This value can be used as a surrogate for the measurement of the repression level of *I*-factor activity in the germ-line, a high fertility level being associated with high repression.

Interestingly, the rate of *I*-factor repression in the SF female germ-line largely depends on the repression capacity already pre-existing in their reactive mother's germ-line before the dysgenic cross (*i.e.* before introduction of the *I*-factor). It has been shown that reactive females naturally acquire an increased repression ability, either during the ageing process or upon a heat treatment [20]. This protection is reversible and transmitted from the reactive mothers to their SF daughters - which become more fertile. The transmission to the next generation and reversibility are typical traits of an epigenetic phenomenon. The capacity of a reactive mother to repress *I*-factor activity can be estimated by measuring the *I*-factor repression level in her SF daughters (obtained after a dysgenic cross). Modifications in the ability to repress the *I*-factor can also be transmitted through several generations and always remain fully reversible [20], [21]. This illustrates the great plasticity of this regulatory system and makes the I-R hybrid dysgenesis syndrome a very good model to study transgenerational epigenetic inheritance.

Previous studies [22]–[26] have shown that prior introduction, into a reactive genome, of transgenes containing an internal region of the *I*-factor, can epigenetically repress-by homology-dependent gene silencing (HDGS) [27]–[29]-the activity of functional *I*-transposons, subsequently introduced by crossing. The protection level increases with the copy number of the regulating *I*-related transgene, whose transcription, but not translation, is required to have an effect [23], [24]. These results suggest that *I* sequence-containing RNA species transcribed from the transgenes are most likely the molecular effectors mediating this epigenetic protection through an RNA interference-driven process.

Several genetic analyses suggest a possible involvement of the pericentromeric non-coding *I*-RE sequences in the natural repression of *I*-factor activity [14], [18], [30], but there is as yet no compelling molecular evidence for a role of the *I*-REs in the epigenetic control of active *I*-factors. Considering our previous results obtained with *I*-related transgenes [18], [22]–[24], and since some of the natural *I*-RE sequences appear to be transcribed in some conditions [31], it was of interest to determine whether *I*-RE RNAs could be naturally protective molecules against *I*-factor invasions.

To test this hypothesis, we compared in the ovaries of reactive females, during ageing and upon heat treatment, the amount of *I*-RE transcripts (measured by quantitative real-time RT-PCR) and the ability to repress *I*-factor activity (assessed in the dysgenic SF progeny, after introduction of the *I*-factor by crossing, as described in reference [20]). We also compared both parameters between the ovaries and the bulk of the other tissues, in which the *I*-factor is normally silent. We found evidence for a role of *I*-RE RNAs in the natural protection against new invasions by *I*-factors. Furthermore, our results provide insight about a specific transcriptional regulation of the heterochromatic *I*-REs. We also discuss the nature of the mechanism involved in *I*-factor repression by the *I*-RE transcripts, and of the epigenetic “imprint” that can be transmitted through several generations.

## Results and Discussion

### Influence of Ageing on the Ovaries of Reactive Females

The ability to repress *I*-factor activity in the ovaries of ageing reactive females, which is transmitted to the progeny, was assessed by measuring the level of *I*-factor repression in their SF daughters (as in reference [20], equated with the level of fertility of SF females of constant age, *i.e.* the percentage of hatching eggs they lay). Relative amounts of *I*-RE transcripts were measured in the ovaries of reactive females (*i.e.* the mothers of SF females) at different ages, by performing quantitative real-time PCR after random reverse transcription of total RNA (RT-PCR). In order to detect a maximum of *I*-RE transcripts, the primer pair used for quantitative PCR was designed so as to match with at least six different *I*-RE sequences that had been determined previously [17], [18] (accession numbers for Ip2918, Ip3172, Ip2862 and Ip3036 in [18], GenBank accession numbers DQ988686 and DQ988685 for I503 and I507 respectively) and was checked for successful amplification of a wide range of *I*-REs, by sequencing fifteen products from a PCR performed on genomic DNA or on reverse-transcribed total RNAs. We found eight different sequences: two of them matched two of the *I*-REs that had been used to design the primers, and six sequences corresponded to other *I*-REs, showing that indeed a set of divergent *I*-RE sequences can be amplified.

**Figure 1:**
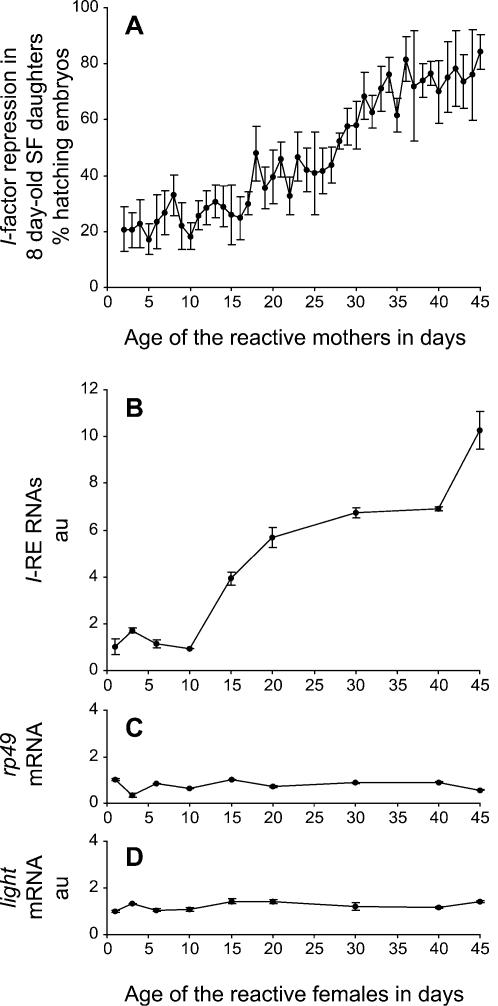
*I*-factor Repression in SF Daughters Correlates with *I*-RE Transcript Levels in their Ageing Reactive Mothers. (A) Ability to repress the *I*-factor, which pre-exists in the ovaries of ageing reactive mothers, was assessed after a dysgenic cross with young *w^1118^* inducer males, by measuring the level of *I*-factor repression in their SF daughters, *i.e.* the percentage of hatching embryos they laid at constant age (8 day-old). The age of the *w^K^* reactive mothers used for the dysgenic crosses is indicated. Relative amounts of *I*-RE RNAs (B) as well as control *rp49* (C) and *light* (D) mRNAs, were measured by performing specific quantitative real-time PCR on randomly reverse-transcribed total RNAs from ovaries dissected from *w^K^* reactive flies at different ages. Values were normalized to 18S rRNA levels. au, arbitrary unit. The age of the *w^K^* reactive females is indicated. Bars represent standard deviation from the mean.

**Figure 2:**
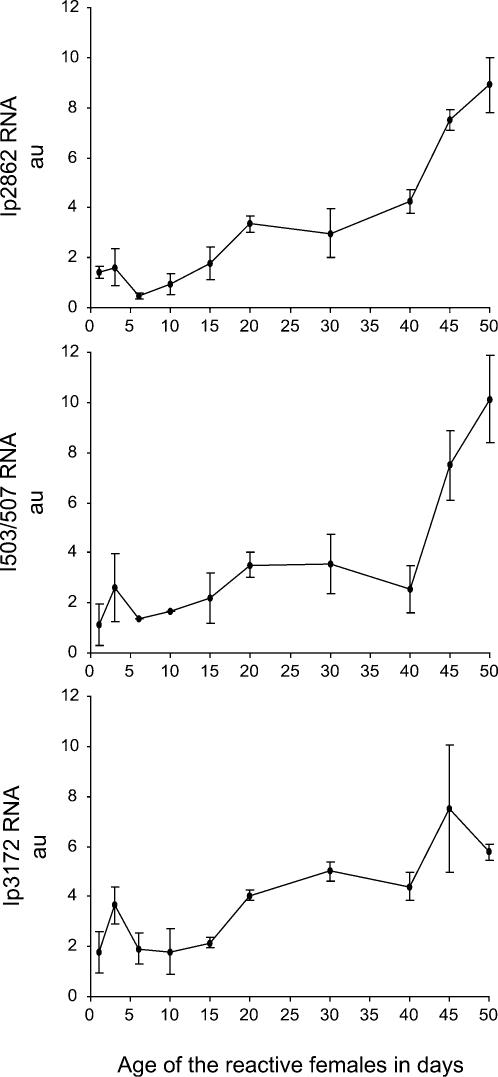
The Amount of Transcripts from Individual *I*-REs Increases in Reactive Ovaries during Ageing. Relative amounts of transcripts from different *I*-REs (named Ip2862, I503/507 and Ip3172) were individually measured by performing specific quantitative real-time PCR (as described in the legend to Figure 1). I503/507 represents the measurement of two *I*-REs (I503 and I507) which could not be differentiated by the primers used for PCR.

We found a very similar increase for both the amount of *I*-RE transcripts in the ovaries of ageing reactive flies and the magnitude of *I*-factor repression in their SF daughters (Figure 1). The amount of *I*-RE RNAs (Figure 1B) increased by a factor of ten in the ovaries of reactive females between their first and their forty-fifth day of life, while the ability to repress the *I*-factor (Figure 1A) rose in parallel from a minimum of 17% during the first days of life, to an almost fully protective value of 84% at day 45. This excellent correlation was the first piece of evidence for a role of the *I*-RE RNAs in the capacity of a reactive genome to repress *I*-factor activity. An increase, by factors of 3, 6.5 and 9, was also observed between one day-old and fifty day-old reactive females, for RNAs from three specific *I*-REs (Ip3172, Ip2862 and I503/507, respectively) tested individually by quantitative real-time RT-PCR (Figure 2), using sequence-specific primers. This suggests that many, if not all, *I*-RE transcripts individually behave the same way, despite the *I*-REs being scattered at distinct locations in the Drosophila genome. These effects do not appear to be due to some general phenomenon affecting the genome globally or involving specifically the pericentromeric heterochromatin, as both control mRNAs from the *rp49* gene (located in euchromatin, EMBL-Bank accession number X00848) and the *light* gene (located in pericentromeric heterochromatin, GenBank accession number AF034571) displayed no significant variation (Figure 1C and 1D). These results suggest that the increase in the amount of *I*-RE RNAs observed during ageing could be the consequence of a transcriptional activation event affecting specifically the *I*-REs or at least the heterochromatic domains where they are located. The specific increase in *I*-RE transcripts could also be the consequence of RNA accumulation due to a stabilizing effect, but other results were not consistent with such an hypothesis (see below).

### Influence of Heat Treatment on the Ovaries of Reactive Females

**Figure 3:**
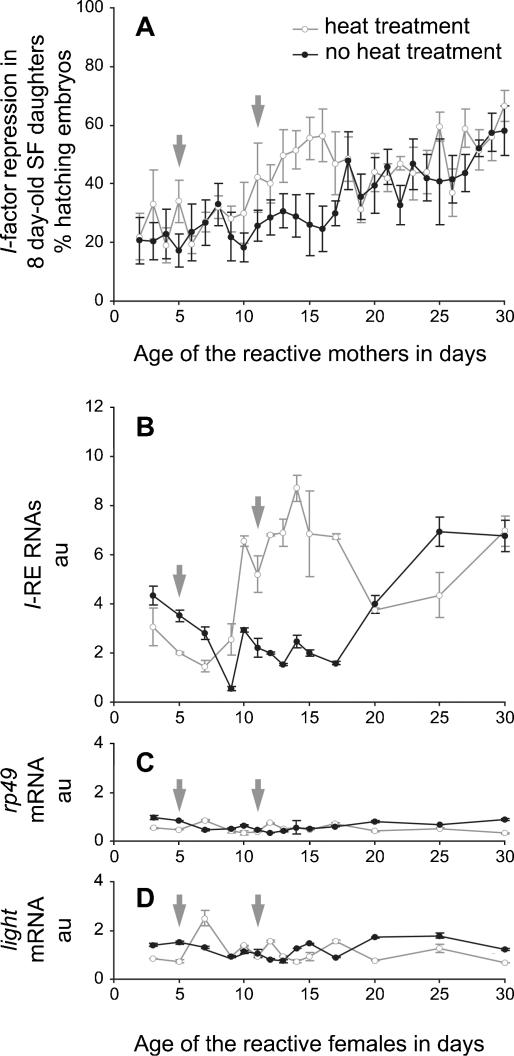
*I*-factor Repression in SF Daughters Correlates with *I*-RE Transcript Levels in their Heat Treated Reactive Mothers. Ability to repress the *I*-factor (A) and the relative amounts of *I*-RE RNAs (B) as well as control *rp49* (C) and *light* (D) mRNAs were measured as described in the legend to Figure 1, but using ageing reactive females/mothers subjected or not to a 31°C heat treatment, flies being normally reared at 22°C. Gray arrows indicate the beginning and the end of the 31°C heat treatment.

We measured the ability to repress *I*-factor activity and the amount of *I*-RE RNAs in ovaries from reactive females subjected or not to a 31°C heat treatment between days 5 and 11, the normal rearing temperature being 22°C (Figure 3). Once again, the level of *I*-repression observed in SF females (Figure 3A) followed precisely the same kinetics as the amount of *I*-RE RNAs in their reactive mothers (Figure 3B), confirming that these transcripts could be implicated in the natural protection against *I*-factor activity. Compared to the controls without heat treatment, both the *I*-repression (in the SF progeny) and the *I*-RE transcript levels (in the reactive mothers) displayed a transient, reversible increase that started after the same delay of four days following the rise in temperature from 22 to 31°C (at day 9), and ended more than six days after the end of the treatment (after day 17).

The RNA profile (Figure 3B) is not compatible with a mere stabilization of *I*-RE RNAs. The sharp increase caused by heat treatment (days 9–10) suggests that RNA synthesis is actively stimulated compared to the control. The sharp decrease-back to a level equivalent to that of untreated flies-following the effect of the heat treatment (after day 17) argues against long-term RNA stabilization. These observations indicate that *I*-RE RNAs are rather unstable and that their level is likely controlled by the rate of transcription.

Long delays were observed for both the increase in the amount of *I*-RE RNAs after the start of exposure to heat (four days), and the beginning of its decrease after the end of the treatment (more than six days). Such delays are not consistent with a “classical” response to heat shock (which is much faster), suggesting that these intervals are necessary for other mechanisms first to take hold and then to die down. This is consistent with the involvement of epigenetic mechanisms in the control of *I*-RE transcription (see Concluding Remarks).

As observed in the case of ageing, the effect of heat treatment on *I*-RE transcription is unlikely to be due to global de-repression of gene expression, since the mRNAs from both the *rp49* and *light* genes did not display any significant variations (Figure 3C and 3D).

### Comparison of the Amount of *I*-RE RNAs between the Ovaries and the other Tissues of Reactive Flies

**Figure 4:**
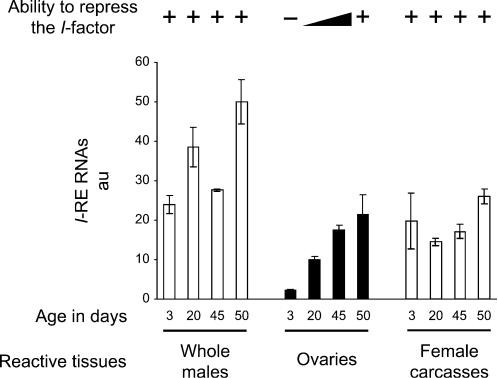
Ability to Repress the *I*-factor Correlates with the Amount of *I*-RE Transcripts in Tissues from Ageing Flies. Relative amounts of *I*-RE RNAs were measured (as described in the legend to Figure 1) in whole males, ovaries and carcasses of females (devoid of ovaries) from w^K^ reactive flies of different ages. Ability to repress the *I*-factor in each tissue is schematized (+, high repression ability; -, low repression ability).

We measured the relative amounts of *I*-RE RNAs in whole males, dissected ovaries and carcasses of females (tissues remaining after ovary dissection) from ageing reactive flies (Figure 4). Once again, a strong correlation between the amount of *I*-RE transcripts and the ability to repress *I*-factor activity was observed, further supporting a regulatory role for these RNAs. The progressive increase in the amount of *I*-RE RNAs in the ovaries (already observed in Figure 1B) was confirmed. Furthermore, during ageing, ovarian *I*-RE RNAs tend to reach the “threshold” level found in the bulk of tissues known to be constitutively repressive for *I* transposition (whole male flies and carcasses of females), which constantly produce a higher (or at least equal) amount of *I*-RE RNAs than the ovary from old reactive females. This high level of transcripts in non-permissive tissues suggests that the *I*-RE RNAs could also play a role in the capacity of tissues other than the ovaries to repress *I*-factor activity, *e.g.* somatic tissues, where the regulation of the *I*-factor has been poorly investigated. Thus, the difference of regulation between somatic tissues and the female germ-line could simply be the consequence of quantitative variations in the regulatory RNAs produced by *I*-REs.

### Concluding Remarks

We show here the activation of multiple non-coding heterochromatic elements, during ageing and upon heat-treatment, in the *Drosophila melanogaster* female germ-line. These sequences, located in the pericentromeric region of chromosomes, are the remnants of an ancestral invasion(s) by a transposable element related to the *I*-factor. We show a strong correlation between the transcript level of these ancestral *I*-related elements (*I*-REs), measured in the ovaries of ageing or heat-treated reactive mothers, and the repression level of the functional *I*-factor, measured in the germ-line of their SF daughters (obtained by crossing the ageing or heat-treated reactive mothers with males containing functional copies of the *I*-factor). High *I*-RE transcript levels were also found in the female soma and in whole males, where permissiveness to *I* activity is very low. These results are consistent with a role of the *I*-REs in the natural transgenerational repression of *I*-factors. This also suggests that the molecular basis of *I*-factor regulation could be the same in the ovary and in the bulk of the other tissues. The difference would be that the ovary can modulate the amount of *I*-RE transcripts, thus repressing or allowing *I*-factor activity depending on the circumstances, whereas the other tissues constitutively produce a sufficient amount (above a threshold level) of these RNAs to be always repressive. Thus, fly individuals are protected from the deleterious effects of transposition, while leaving a possibility for the *I*-factor to transpose in the female germ-line. Such a mechanism could have been selected to maintain a balance between the stabilization of the genome and the need to create variations for the sake of genetic diversity. These variations arising in germ cells within the ovary would be inherited and could thus play a role in the process of adaptive evolution.

We had previously shown that transgenes containing a fragment of the *I*-factor can efficiently silence *I* activity. Such transgenes have to be transcribed, either in sense or anti-sense orientation, but a coding region is not required for *I* silencing [22]–[24]. These data show that the introduction of additional transcribable *I*-like sequences (which can be considered as equivalent to the natural *I*-REs) leads to *I*-factor silencing, by homology-dependent gene silencing (HDGS). Moreover, such transgenes induce the same characteristic epigenetic traits as those observed for *I*-factor silencing under natural conditions, during ageing or upon heat-treatment, *i.e.* maternal inheritance, transmission over generations and reversibility of the silencing effect. We have also provided evidence that cosuppression between non-homologous *I*-related transgenes (one containing the *I* promoter, and the other containing an internal fragment of the *I*-factor) follows the same rules of heredity and reversibility. These data suggest that the natural *I*-REs (already present in the genome and containing sequences homologous to both transgenes) could have a key regulatory role in the silencing of *I*-like sequences (as an “intermediary” between the two transgenes in this case) [18].

In the light of the results presented here, these former data, obtained with transgenes, thus appear to be an experimental reproduction of the natural process of *I*-repression by *I*-REs. As *I*-REs do not encode any protein, they probably protect the *Drosophila melanogaster* genome against *I*-factor invasion through the RNA-mediated process of HDGS. This is consistent with: (i) the level of nucleotide identity between *I*-REs and *I*-factors, which is above 91% [17], [18], considering that 90% of nucleotide identity is sufficient to trigger RNA-mediated silencing in Drosophila [32]; (ii) the presence of both sense and anti-sense transcripts from *I*-REs in the ovary of ageing reactive flies (our unpublished results), suggesting that double stranded RNAs can be generated to trigger RNA interference; and (iii) the fact that mutations in genes involved in RNA interference pathways can lead to an increase in the amount of *I*-like RNAs in inducer strains; however, it is unknown whether these *I*-like RNAs originate from the *I*-REs and/or the functional *I*-factor [33], [34].

The *I*-factor is a transposon similar to mammalian LINEs. In agreement with our hypothesis that LINE transposons can be silenced by homologous RNAs, Yang and Kazazian have recently provided evidence for the processing of double stranded RNAs from LINEs into silencing small interfering RNAs in human cultured cells [35]. Repeat-associated small interfering RNAs (rasiRNAs) are short regulatory RNAs homologous to repeat sequences. rasiRNAs complementary to LINEs and other transposons have been found in mouse oocytes [36], in Drosophila [37] and in several other eukaryotes (reviewed in reference [38]). One essential question that remains to be answered is the origin of rasiRNAs. Even though rasiRNAs homologous to the *I*-factor have not been detected in a search for Piwi-associated rasiRNAs in Drosophila [37], our data suggest that rasiRNAs complementary to transposons (at least to LINEs) might originate from the bulk of defective, mostly non-coding elements, which would have been conserved in order to silence the corresponding functional mobile elements.

Concerning the up-regulation of *I*-REs expression, especially following heat-treatment, our data show evidence for specific regulation by a mechanism distinct from that involved in the classical heat shock response. A hypothetical mechanism can be proposed based on the results previously reported by A. Bucheton et al. [14]. They showed that the introduction by transgenesis of an additional copy of the *Su(var)3-9* gene resulted in a decrease in the ability of the reactive female germ-line to repress the *I*-factor. The product of *Su(var)3-9* is a key partner of HP1 (Heterochromatin Protein 1) involved in the condensation of chromatin, notably in the pericentromeric region [39]. Thus, the transcription of the *I*-REs could be controlled by changes occurring at the level of their chromatin structure, under the influence of proteins such as that specified by *Su(var)3-9*, and its molecular partners. What might drive the specific targeting of *I*-REs by such a process remains to be determined.

Modifiers of chromatin structure are also involved in the stable transmission of epigenetic “marks” across cell generations at the level of heterochromatin [39]. This could explain how the ability to repress *I* activity is transmitted from reactive mothers to their SF daughters after a dysgenic cross, and how it can be further transmitted through several generations. Thus, the nature of the maternal “imprint” is more likely to be an epigenetic transmission of information determining a transcription level through a stabilized chromatin structure (controlled by specialized proteins such as the product of *Su(var)3-9*) rather than a direct transmission of the RNA molecules, which are presumably unstable. Another possibility would be a transmission via small regulatory RNAs, such as rasiRNA derived from the long *I*-RE RNAs. The two mechanisms are not mutually exclusive, especially as small RNAs have been found to be involved in heterochromatin assembly (reviewed in reference [40]).

In conclusion, this work provides evidence for a role of RNAs encoded by defective remnants of ancestral transposon invasions (the *I*-REs) in protecting a genome against the highly mutagenic effects of functional transposable elements (the *I*-factors). Since heterochromatic *I*-REs are the “memory” of ancestral invasions by *I*-factor-like transposons, this protective process, most probably involving an epigenetic mechanism of natural RNA-mediated HDGS, can be considered as a genetic “vaccination” against transposable elements. It is noteworthy that *I*-REs are vestiges of a transposable element related but not identical to the *I*-factor. The consensus of the ancestral element is divergent from the *I*-factor by 4 to 5% at the level of the nucleotide sequence (our unpublished data), indicating that this protection could tolerate some divergence between the ancestral elements and the functional invading transposon to be silenced. Such a mechanism is likely to be widespread throughout the eukaryotic kingdom and for all classes of transposable elements because: (i) remnants of mobile elements are a major component of most eukaryotic genomes, notably that of humans, at least 45% of which is composed of such sequences, with 20% related to LINEs [11], (ii) the repression mechanism of transposable elements from different classes shows striking similarities with *I*-regulation [41]–[44], and (iii) when they are still functional, transposons are generally “tamed” by their host.

Last but not least, the present study suggests that *I*-RE transcripts are the molecular determinants of the so-called “level of reactivity” (defined as the permissiveness to *I*-factor activity in the reactive female germ-line) [14] in the I-R system of hybrid dysgenesis, which had remained a mystery for several decades.

## Materials and Methods

### Drosophila strains

The *w^1118^* inducer strain [45], which contains functional *I*-factors, and the *w^K^* reactive strain [46], which does not, were gifts from D. Coen and C. McLean. Flies were reared on standard medium at 22°C±1°C.

### Heat treatment

For the heat treatment, the incubation temperature of 5-day-old flies was raised from 22°C to 31°C, kept at 31°C for 6 days, before being lowered back to and maintained at 22°C until the end of the experiment.

### Measurement of the ability to repress the *I*-factor in the ovaries of reactive females

Several groups of virgin *w^K^* reactive females were crossed en masse with young *w^1118^* inducer males. A proportion of these groups was maintained permanently at 22°C. The other part was subjected to the heat treatment described above. *w^1118^* males were replaced by young ones every week. Even during the heat treatment, eggs were collected every 24 hours and then kept at 22°C. The first 20 SF females born from each batch of eggs (laid by reactive mothers of different ages, subjected or not to the heat treatment) were allowed to mate with their brothers. At a constant age (when 8-day-old), these SF flies were transferred to an egg collector. Sixteen hours later, five to ten batches of 40 eggs were deposited as 4×10 matrices, thus allowing unambiguous counting (a further 48 hours later), of hatched and non-hatched (dead) embryos. The percentage of hatching embryos laid by SF females, which corresponded to the level of SF fertility, was used as a measurement of *I*-repression in their ovaries. This value was also used as a surrogate for the assessment of the ability to repress the *I*-factor that pre-existed in the ovaries of their reactive mothers.

### Quantitative real-time RT-PCR

Total RNAs were extracted with TRI Reagent^®^ (Sigma) from 50 *w^K^* males, 50 carcasses or 50 pairs of ovaries dissected from *w^K^* females of different ages, subjected or not to the heat treatment described above. Quality of the extracted RNAs was assessed on an RNA LabChip^®^ (Agilent 2100 Bioanalyzer), and RNA concentration was determined spectrophotometrically. Twenty micrograms of each RNA sample were subjected to DNase treatment (DNA-free; Ambion). One microgram of RNA from each sample was reverse-transcribed in a 20-µl reaction using 50 U of Moloney murine leukemia virus RT and 20 U of RNuclease inhibitor (Applied Biosystems), 1 mM each of dATP, dTTP, dGTP and dCTP (Amersham-Pharmacia Biotech), 5 mM of MgCl_2_, 10 mM of Tris-HCl (pH 8,3), 10 mM of KCl, and 2.5 µM of random hexamers (Applied Biosystems). The cDNAs were diluted 1/20 in nuclease-free water. Real-time quantitative PCRs were then achieved with 5 µl of each cDNA dilution, in a total volume of 25 µl, using SYBR^®^ Green PCR Master Mix, or TaqMan^®^ Universal PCR Master Mix (both from Applied Biosystems) for the detection of 18S rRNA. Amplifications were performed with the ABI PRISM^®^ 7000 sequence detection system (Applied Biosystems), using a 2-min step at 50°C and then a 10-min denaturation step at 95°C, followed by 40 cycles of 15 sec of denaturation at 95°C and 1 min of primer annealing/polymerization step at 60°C. The relative expression between different samples was calculated with respect to a standard calibration curve (a dilution series of genomic DNA). To normalize for differences in the amount of total RNA added to the reaction, measurement of 18S rRNA was performed as an internal control. The primers and probe for 18S were purchased from Applied Biosystems. The primers (sequence available upon request) for the whole *I*-REs, individual *I*-REs (Ip2862, I503/507 and Ip3172), *light* and *rp49* were designed with the computer program Oligo (Medprobe), and purchased from MWG Biotech.
